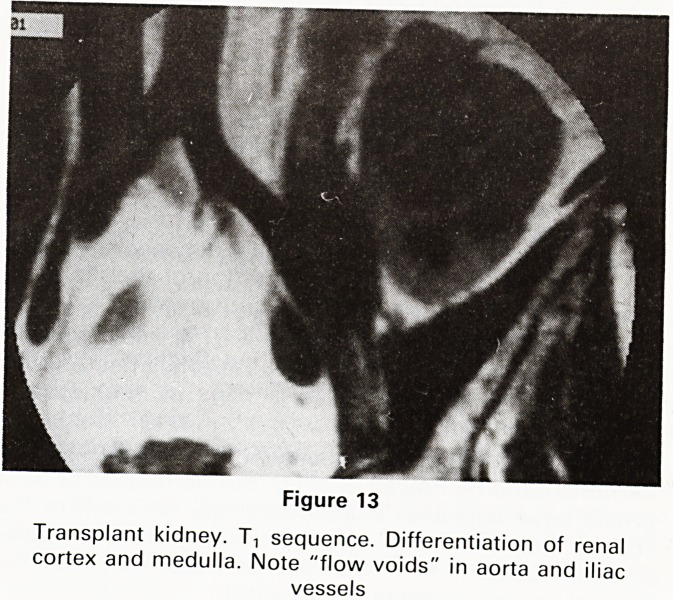# MRI and the District General Hospital

**Published:** 1988-05

**Authors:** C. Johnson, J. B. Penry

## Abstract

Experience at Southmead Hospital confirms the importance of magnetic resonance imaging as a non-invasive method of studying the central nervous system. Its usefulness in the investigation of other systems, notably the genito-urinary tract and bones, joints and soft tissues is, however, stressed. It is a continually developing modality and the probability is that its applications will become even more widespread in the future.


### MRI and The District General Hospital

**C. Johnson, BCh, DMRD, FRCR and J. B. Penry MB, BCh, DMRD, FRCR**
Based on a paper read (CJ) to The Bristol Medico-Chirurgical Society on 14th January 1988

### ABSTRACT

Experience at Southmead Hospital confirms the importance of magnetic resonance imaging as a non-invasive method of studying the central nervous system. Its usefulness in the investigation of other systems, notably the genito-urinary tract and bones, joints and soft tissues is, however, stressed. It is a continually developing modality and the probability is that its applications will become even more widespread in the future.

### INTRODUCTION

Long before the Bristol Magnetic Resonance Scanner came on the scene, neurologists, neurosurgeons and neuroradiologists were well aware of its potential, indeed its proven worth in the investigation of disorders of the central nervous system. (1,2) As a District General Hospital Southmead generates a fair amount of investigative neurology and it is true to say that if one includes the investigation of the spine, then neurology provides the largest single source of cases investigated by magnetic resonane (MR) at Southmead, we have been very impressed by its usefulness in this respect.

In this presentation, however, we deal with cases from sources other than neurology in which MR has proved of value. Most of the hospital departments have been involved and the cases presented therefore are a very heterogeneous collection.

### THE UROGENITAL TRACT

**The Prostate Gland**

Benign hypertrophy is well shown on $T_1$ weighted images in the sagittal projection, the enlarged gland giving a uniform signal and being separated from surrounding pelvic structures by both the high signal from pelvic fat and the flow voids of the prostatic venous plexus (Figure 1).

**Figure 1**

Benign prostatic hypertophy. $T_1$ sequence showing high signal (white) between gland and rectum

The ultimate aim of MR of the prostate, as with rectal ultrasound, must be the diagnosis of small malignancies before they have breeched the capsule of the gland. We have not yet been able to do this with any degree of certainty and other centres vary in their claims in this respect. (3,4) We have therefore been mainly concerned with demonstrating extraglandular spread and the complications of carcinoma of the prostate. The extent of intravesical spread of a malignant prostate (Figure 2) can be shown, as can lymphadenopathy (Figure 3).

In this respect the investigation is complimentary to computerised tomography.

**Figure 2**

Prostatic carcinoma. $T_1$ sequence showing intravesical spread and loss of fat line between gland and rectum

**Figure 3**

Carcinoma of prostate. $T_1$ sequence showing lymphadenopathy adjacent to right acetabulum (arrowed)

### The Urinary Bladder.[5]

Bladder tumours are, of course, diagnosed by clinical history and examination, conventional radiology and endoscopy. MR can, however, demonstrate the degree of infiltration of the bladder wall (Figure 4) and local spread into the pelvis. In a case illustrated (Figure 5) the relationship of the bladder tumour to a bladder diverticulum was a matter of doubt at endoscopy. It was felt that the MR images showed the tumour reaching the edge of, but not entering, the diverticulum.

**Figure 4**

Carcinoma of bladder. STIR (short tau inversion recovery) sequence highlighting sessile polypoid tumour arising from and infiltrating left wall of bladder

**Figure 5**

Carcinoma of bladder. $T_1$ axial sequence; double polypoid tumour reaching edge of diverticulum of bladder

### Ectopic Testes

$T_1$ weighted images in the coronal and axial planes give an excellent demonstration of the site and size of intra-abdominal ectopic testes (Figure 6).

**Figure 6**

$T_1$ coronal series. Intrapelvic ectopic testes either side of bladder (arrowed)

### THE MUSCULO-SKELETAL SYSTEM

### The MR myelogram

Cases of known prostatic malignancy present not infrequently at Southmead Hospital with disturbances of gait or even frank paraplegia. Plain radiographs will often demonstrate bony metastatic disease as will an isotope study but hitherto myelography has been necessary to document spread of tumour tissue into the spinal canal. The MR MAST (motion artefact suppression technique) facility enables us to obtain images which are virtually identical with those obtained by conventional radiographic myelograms, the difference being, of course, that with MR no intrathecal injection is necessary (Figures 7 and 8). Only one conventional myelogram has been carried out at Southmead Hospital since the advent of the Bristol MR Scanner.

In two recent cases, MR has demonstrated the destruction of the discs and adjacent bone end plates in "discitis". These appearances are, of course, eventually well demonstrated on plain radiographs. The advantage of MR is the ability to show the changes before they are apparent on the radiograph but, more importantly, to demonstrate the extent of any associated soft tissue mass, particularly with reference to any extra-dural collection which might compromise the cord.

**Figure 7**

Metastatic carcinoma of prostate. MAST (motion artefact suppression technique) sequence highlighting vertebral body metastasis but no evidence of spread within the spinal canal

**Figure 8**

Same sequence as figure 7. Vertebral body involvement as before but with evidence of tumour tissue within the canal displacing the cord posteriorly.

**Soft Tissue Masses**

Two cases of clinically similar soft tissue masses in the posterior aspect of the lower thigh were investigated.

In the first, ultrasound examination had suggested a fluid basis with some additional solid elements. The $T_1$ sequence showed a complete absence of signal consistent with a "cyst" and this was confirmed by the very high signal on the $T_2$ series (Figure 9). Aspiration revealed clear fluid and the cyst was presumed to have a traumatic origin. The $T_2$ series of the second case showed a fairly low signal quite unlike the $T_2$ images of the cyst (Figure 10). This was correctly diagnosed as a sarcoma. This degree of specificity is helpful in the accurate diagnosis of disease processes. (6)

**Figure 9**

Thigh "cyst". $T_2$ sequence demonstrating encapsulated fluid

**Figure 10**

Thigh sarcoma. $T_2$ sequence showing relatively low signal from well defined tumour mass

**Knee Arthrography**

Conventional radiographic arthrography of the knee involves the introduction of contrast medium and air into the joint. The latter remain in the joint for several days and may be a source of discomfort or pain to the patient. In addition, there is the small attendant risk of the introduction of infection. Sagittal $T_1$ weighted images (Figure 11) with or without coronal views, now give information regarding semilunar cartilage degeneration and tears and the status of the cruciate ligaments with an accuracy similar to that of conventional arthrography without the necessity for an invasive procedure. (7)

**Figure 11**

$T_1$ sagittal view through the knee joint. Note well demonstrated semilunar cartilages

**Renal Transplant Patients**

Patients who have undergone renal transplantation and who are immuno-suppressed are at risk of developing asceptic necrosis of the femoral heads.

**Figure 12**

$T_1$ sequence (Field Echo). Lack of signal (black) from areas of the femoral heads indicating aseptic necrosis

**Figure 13**

Transplant kidney. $T_1$ sequence. Differentiation of renal cortex and medulla. Note "flow voids" in aorta and iliac vessels

This sometimes requires prosthetic hip replacement. Renal transplant patients are now being examined with a view to the earlier diagnosis of this condition. Three such cases, one bilateral, have so far come to light (Figure 12). Conventional radiographs, computerised tomography and isotope studies have been normal on these patients.

The investigation of the hips of these patients has enabled us to study in addition the transplanted kidneys which are well shown on the coronal images of the hips. The healthy transplant shows a clear differentiation of renal cortex and medulla on the $T_1$ sequences (Figure 13), a feature which is lost in the kidney undergoing rejection.

### COMMENT

Our experience at Southmead Hospital has confirmed the recognised value of MR in the investigation of the central nervous system, including the spinal canal where its use excludes the necessity of intrathecal injection of contrast medium. In addition, however, we recognise that the method has widespread applications outside the central nervous system, notably in the genito-urinary tract, the musculo-skeletal system and in the investigation of the renal transplant patient. It is a rapidly developing imaging modality and signs indicate that it might well become the investigation of choice in the study of the circulatory system with all that implies.

**Acknowledgements**

It is a pleasure to record our thanks for the technical expertise of our radiographers, Ann Case and Joanne Waring and of Allen Wood of Picker International.

### REFERENCES

1. PRANT-ZAWADSKI, M. (1988) Magnetic Resonance: state of the art. MR imaging of the brain. *Radiology* **166**, 1–10.
2. BYDDER, G. M. (1984) Magnetic resonance imaging of the brain. *Radiologic Clinics of North America* **22**, No. 4.
3. BRYAN, P. J. BUTLER, H. E. NELSON, A. D. LIPOMA, J. P. KOPIWODA, S. Y. RESNICK, M. I. COHEN, A. M. HAAGAN, J. R. (1986) Magnetic resonance imaging of the prostate. *AJR* **146**, 543–548.
4. HEIKEN, J. P. LEE, J. K. T. (1988) MR imaging of the pelvis. *Radiology* **166**, 11–16.
5. AMENDOLA, M. A. GLAZER, G. M. GROSSMAN, H. B. AISEN, A. M. FRANCIS, I. R. (1986) Staging of bladder carcinoma: MRI-CT-surgical correlation. *AJR* **146**, 1179–1183.
6. SARTORIS, D. J. RESNICK, D. (1987) MR imaging of the musculo-skeletal system. *AJR* **149**, 457–467.
7. BELTRAN, J. NOTO, A. M. MOSURE, J. C. WEISS, K. L. ZOELZER, W. CHRISTOFONDIS, A. J. (1986) The Knee. Surface coil MR imaging at 1.5T. *Radiology* **159**, 747–751.

## Figures and Tables

**Figure 1 f1:**
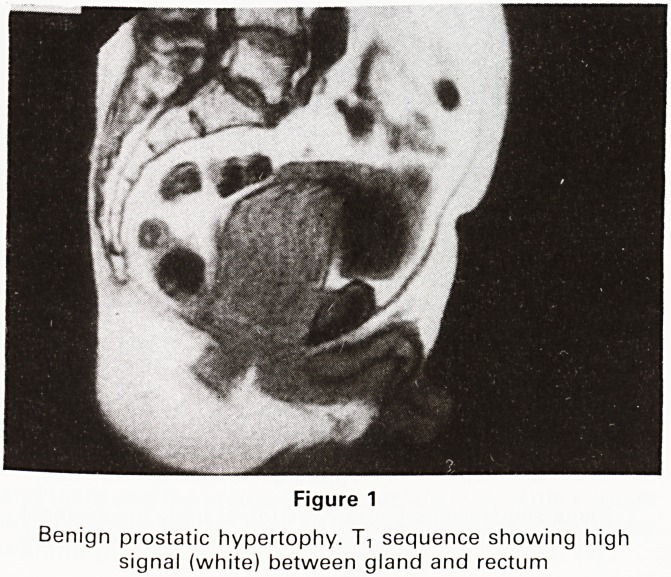


**Figure 2 f2:**
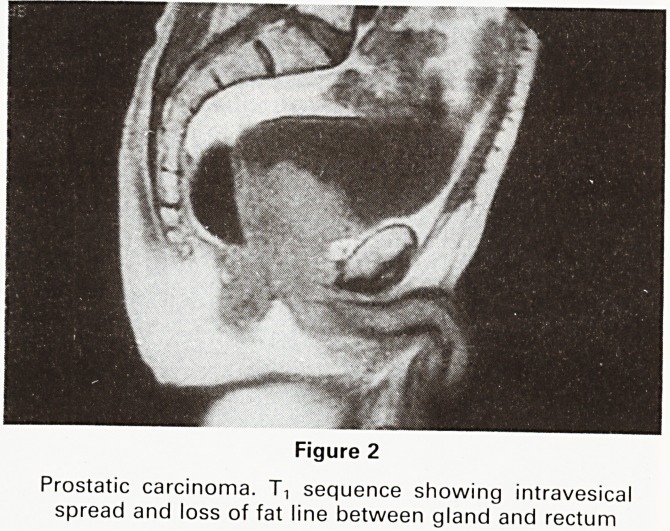


**Figure 3 f3:**
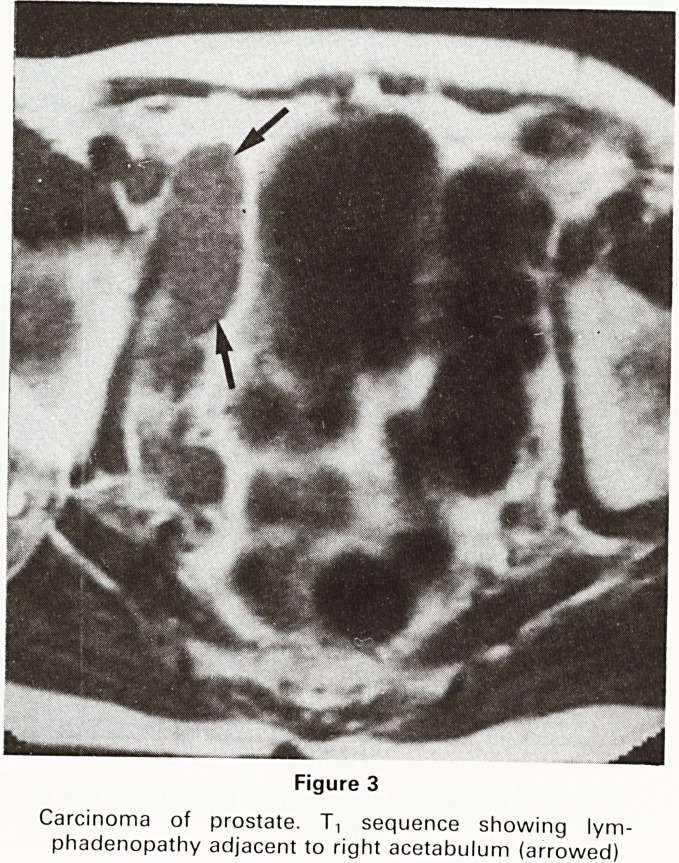


**Figure 4 f4:**
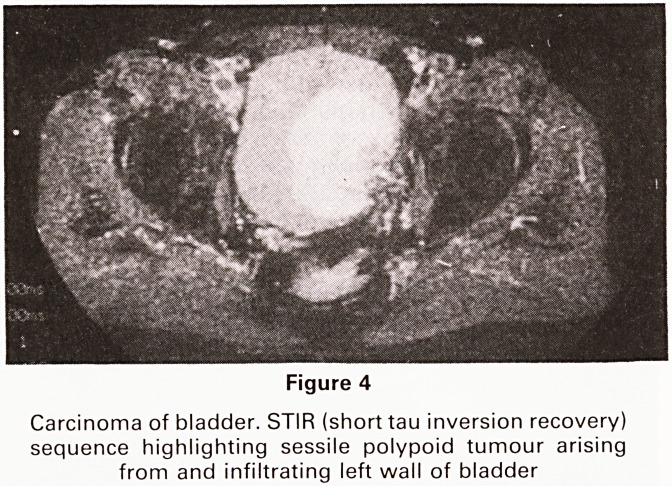


**Figure 5 f5:**
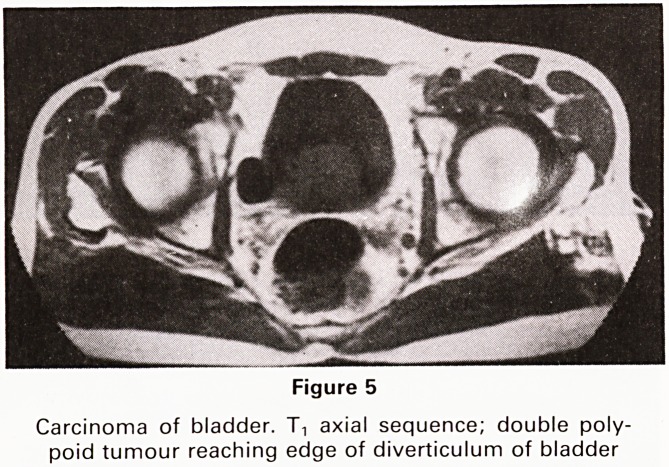


**Figure 6 f6:**
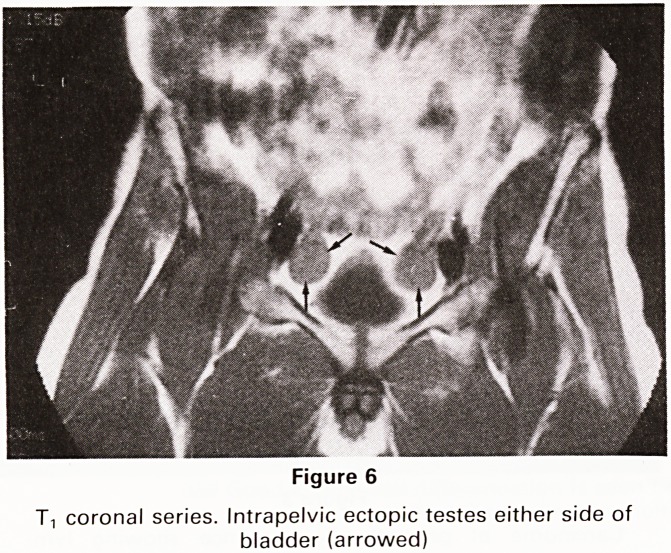


**Figure 7 f7:**
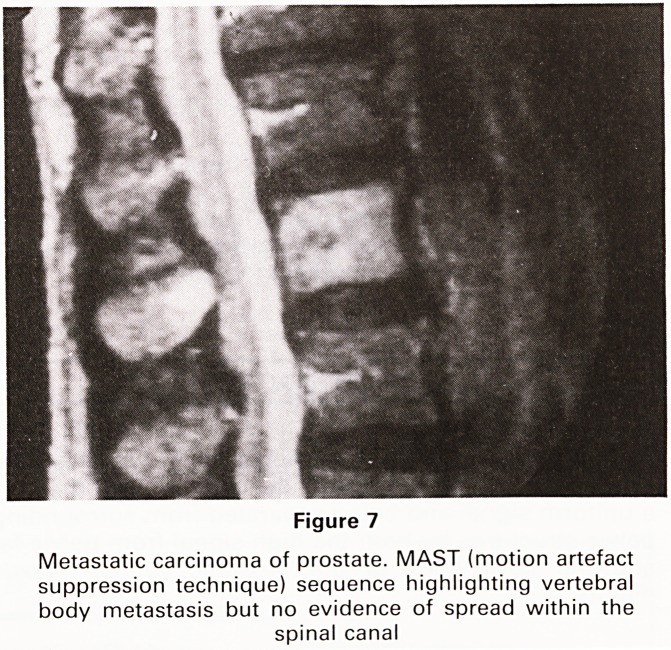


**Figure 8 f8:**
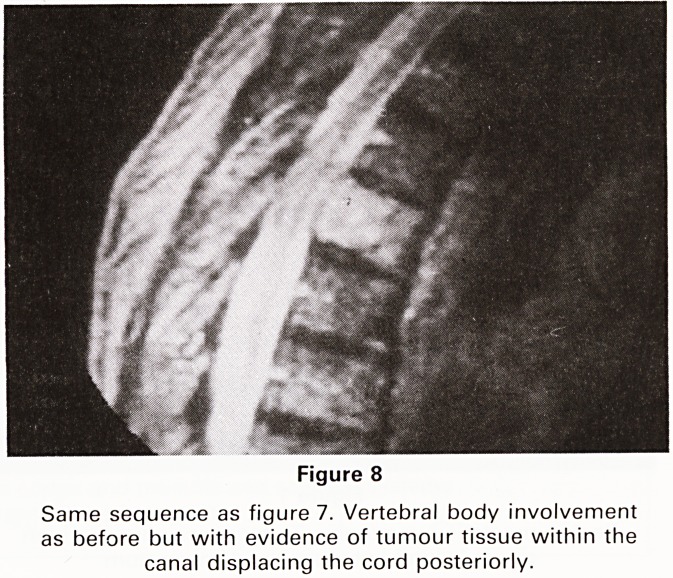


**Figure 9 f9:**
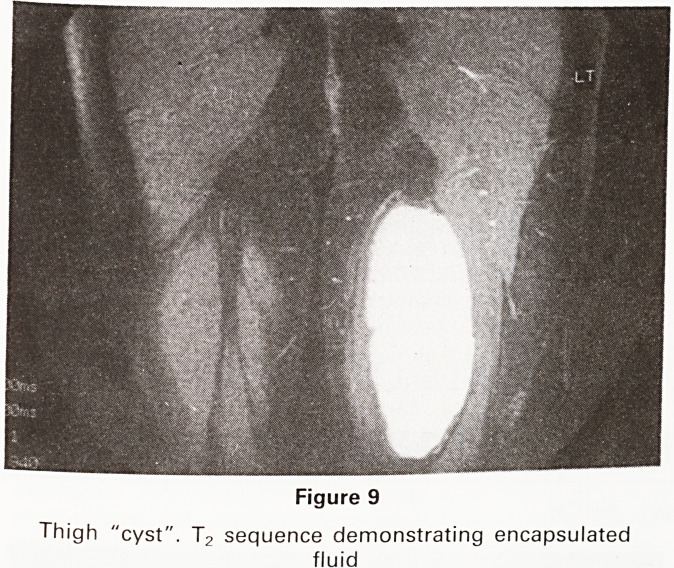


**Figure 10 f10:**
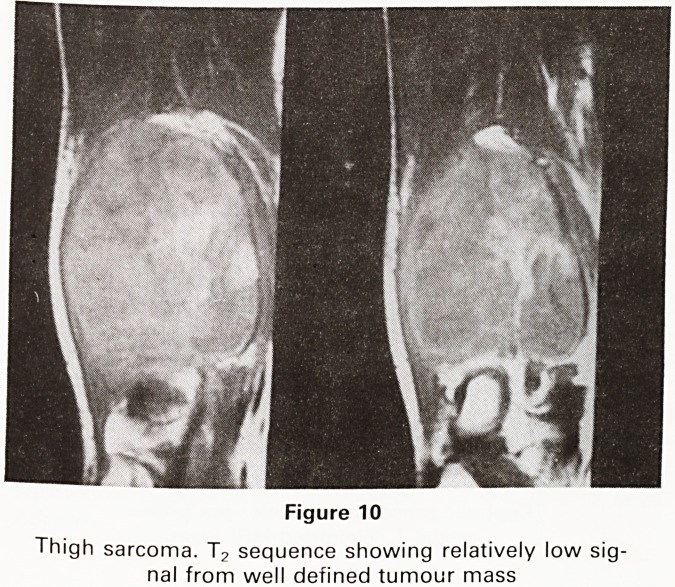


**Figure 11 f11:**
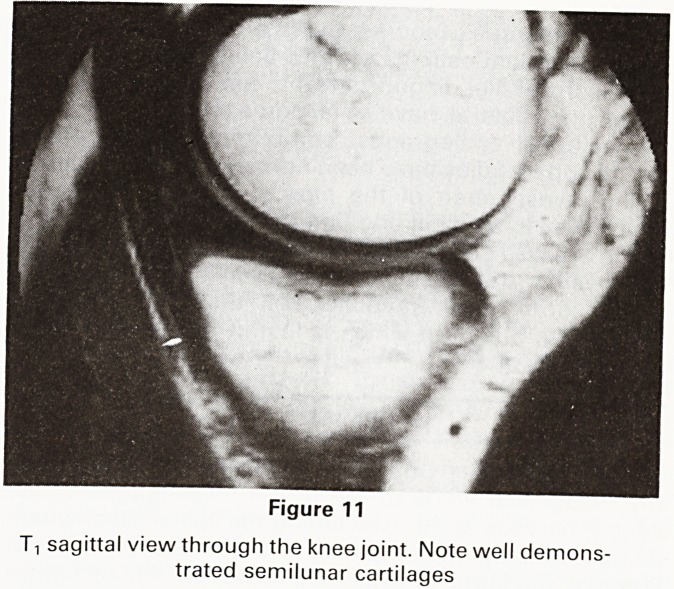


**Figure 12 f12:**
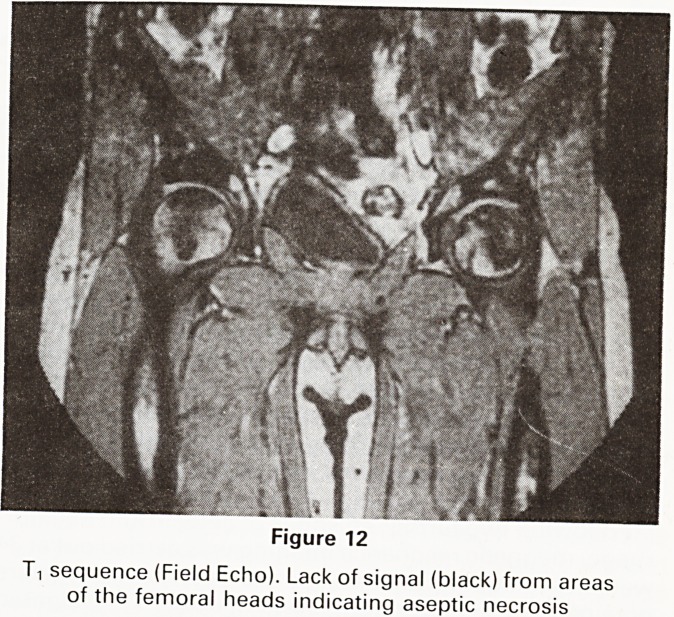


**Figure 13 f13:**